# Case Report: Concurrent primary CNS lymphoma and meningothelial meningioma - nuances of diagnosis and management

**DOI:** 10.12688/f1000research.17770.1

**Published:** 2019-01-25

**Authors:** Samir Kashyap, Jacob Bernstein, Ira Bowen, Rosalinda Menoni, Dan Miulli

**Affiliations:** 1 Department of Neurosurgery, Riverside University Health System, Riverside, CA, 92555, USA; 2 Department of Neurosurgery, Arrowhead Regional Medical Center, Colton, CA, 92324, USA

**Keywords:** CNS lymphoma, Meningioma, Collision tumor

## Abstract

**Background**: The incidence of two distinct primary intracranial pathologies is an exceedingly rare phenomenon. Although meningiomas are well known to coexist with other primary intracranial malignancies there are only nine reported cases of a meningioma occurring simultaneously with primary CNS lymphoma in the literature. We report a case of a woman who sustained multiple injuries due to two distinct intracranial pathologies, however, lateralizing signs were unrecognized for two weeks prior to her final diagnosis.

**Case Description:** A 64-year-old female with history of diabetes mellitus type 2 initially presented to the Emergency Department, two weeks prior, following a mechanical fall at home resulting in a left bimalleolar fracture. CT imaging revealed a right occipital mass with significant vasogenic edema causing 12mm of midline shift. MRI revealed two distinct homogeneously contrast-enhancing lesions: a right occipital mass with dural-based attachment, as well as a homogenously contrast-enhancing lesion adjacent to the right posterolateral ventricle. FLAIR signal changes were also appreciated and were noted to extend across the corpus callosum, raising concerns for a high-grade glial process. She underwent a right occipital craniotomy with gross total resection of the right occipital mass as well as subtotal resection and biopsy of the second lesion. Final pathology of the extra-axial lesion was found to be meningothelial meningioma and the deep lesion was found to be diffuse large B-cell lymphoma.

**Discussion:** We describe a rare instance of simultaneous meningioma and primary CNS lymphoma that was found to be the underlying cause of a traumatic injury several weeks after the incident. We review the current diagnosis and management nuances in the setting of multiple intracranial oncologic processes.

## Introduction

The incidence of two distinct primary intracranial pathologies is an exceedingly rare phenomenon. The reported incidence of such an occurrence is approximately 1 in a million annually ( Lee *et al*., 2002). Although meningiomas, given their benign and slow-growing nature, are well known to coexist with other primary intracranial malignancies such as glioblastoma, metastases, adenomas, there are only nine reported cases of a meningioma occurring simultaneously with primary CNS lymphoma (PCNSL) in the literature ( Gordon *et al*., 2011; Slowik & Jellinger, 1990). Here, we report a case of a woman who sustained multiple injuries due to two distinct intracranial pathologies, however, lateralizing signs were unrecognized for two weeks prior to her final diagnosis.

## Case presentation

**Figure 1.**
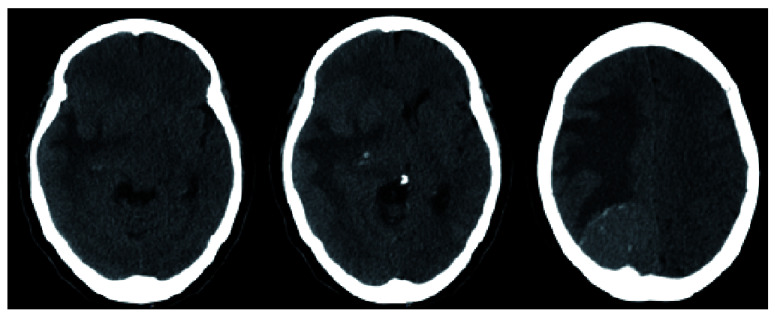
CT head (axial view) demonstrating a calcific right parietal lesion with vasogenic edema as well as a periventricular lesion with associated edema causing 12mm of midline shift.

A 64-year-old Hispanic female with a past medical history of type 2 diabetes mellitus and hypertension presented with a chief complaint of left hemiparesis and paresthesias and was activated as a code stroke. History was limited due to the patient being Spanish-speaking only. She did not receive tPA because she stated her left-sided symptoms were not new and she had progressively worsening clumsiness of her left side and that she had been falling to her left. She presented to urgent care two weeks prior to presentation after sustaining a mechanical fall at home. She was diagnosed with a left bimalleolar fracture, placed in a cast, and scheduled for outpatient follow up with orthopedics for surgical evaluation. Computed tomography (CT) of head revealed a right occipital mass with significant vasogenic edema causing 12mm of midline shift ( Figure 1).

### Clinical exam

The patient was alert and oriented to person, place and time in Spanish. Cranial nerve exam revealed no deficits and no evidence of visual field cut. Motor examination revealed left hemiparesis (4+/5 in the upper and lower extremities), but was limited by the previous casting of her distal left malleolar fracture. Sensory examination revealed slight diminished sensation in the left upper and lower extremities with similar limitations as motor examination.

### Clinical course

**Figure 2.**
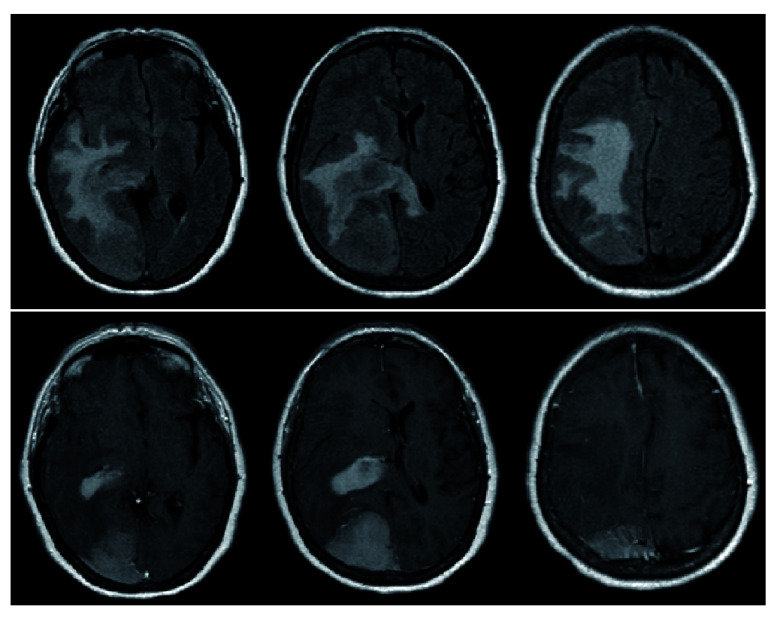
Pre-operative MRI demonstrating diffuse FLAIR changes with evidence of FLAIR signal crossing midline via the splenium of the corpus callosum (top). T1-post contrast reveals 2 distinct lesions – a homogenously enhancing extra-axial lesion in the right parietal lobe as well as a homogeneously enhancing periventricular lesion (bottom).

The patient was started on dexamethasone 6mg every 6 hours and admitted to the ICU. A STAT MRI brain with and without contrast revealed two homogeneously contrast-enhancing lesions: a 4.8.×6.1×3cm right parieto-occipital extra-axial mass with dural-based attachment, as well as a 3.4×1.8×2.2cm homogenously contrast-enhancing lesion adjacent to the right posterolateral ventricle. FLAIR signal changes were also appreciated and were noted to extend across the splenium of the corpus callosum, raising concerns for a high-grade glial process ( Figure 2).

**Figure 3.**
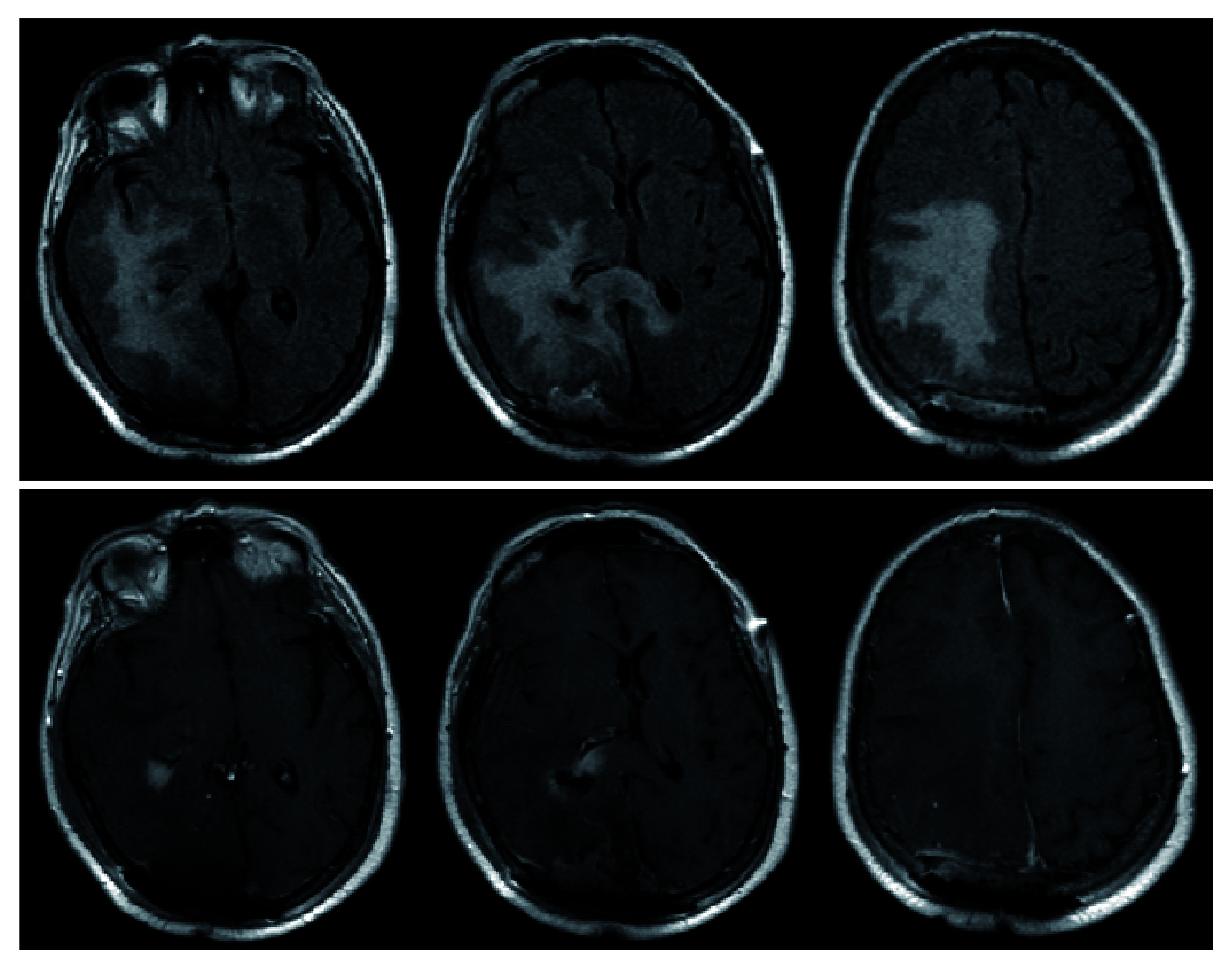
Post-operative MRI demonstrating similar FLAIR changes as pre-operative MRI (top). T1-post contrast reveals gross-total resection of the previously seen extra-axial lesion in the right parieto-occipital region as well as subtotal resection of right periventricular lesion (bottom). The midline shift is significantly improved from pre-operative MRI ( Figure 2).

After preoperative clearance, a right occipital craniotomy was performed with anticipation for gross total resection of the right parieto-occipital lesion and biopsy with likely subtotal resection and biopsy of the second lesion. Preliminary pathology from intra-operative frozen specimen were consistent with meningioma (extra-axial lesion) and high-grade glioma (periventricular lesion). Gross total resection was performed for the extra-axial lesion and maximal, safe resection of the periventricular lesion was performed. She tolerated the procedure well and had an improved neurological exam postoperatively. Her left hemiparesis improved compared with pre-operative exam, however, she did have very minor left visual field deficits. Post-operative MRI demonstrated gross total resection of meningioma and subtotal resection of what was later confirmed to be diffuse large B-cell lymphoma ( Figure 3). During this same admission, she also underwent open reduction, internal fixation (ORIF) of her left bimalleolar fracture without complication. She was discharged home in stable condition.

### Final pathology

Extra-axial lesion: Meningothelial Meningioma 

Periventricular lesion: Diffuse Large B-Cell Lymphoma (+CD20, +BCL-6, +BCL-2, +MUM-1, +KI67)

### Follow-up

At one month clinic follow-up, she was noted to have an intact motor exam with stable visual field deficits on gross examination. She went into complete remission after a course of methotrexate, cytarabine, and Rituxan and 4 cycles of radiation therapy. She tolerated the treatment relatively well with minor symptoms. At one and two year follow-ups, she continues to be in remission with no signs of recurrence on imaging. 

## Discussion

We report a rare case of a concurrent meningioma and primary CNS lymphoma (PCNSL), a rare occurrence entity that has only nine reported cases in the literature. The most common concurrent intracranial tumors reported in the literature are meningioma and glioblastoma ( Zhang *et al*., 2018). It is rare to find two or more primary intracranial tumors simultaneously in patients without previous radiation therapy or underlying phacomatosis such as Neurofibromatosis-2 (NF2). The annual incidence of this phenomenon is estimated to be less than one per million ( Gordon *et al*., 2011; Lee *et al*., 2002).

Accurate diagnosis is essential as the surgical management of these conditions are opposite of one another. One area in which the management in our patient could be improved is a more accurate history and neurological examination. This was likely affected by the fact that the was a non-English speaker and highlights the importance of accurate history taking with a translator to ensure optimal care. Surgical management of PCSNL is typically limited to biopsy if CSF analysis is inconclusive. This is because PCNSL is particularly chemo- and radiosensitive. Conversely, gross total resection is the gold standard in the management of meningiomas and gliomas ( Baraniskin & Schroers, 2014; Gordon *et al*., 2011; Hoang-Xuan *et al*., 2015; Korfel & Schlegel, 2013; Muñiz *et al*., 2014). The same principle applies for steroid administration. The administration of glucocorticoids is not recommended in lymphoma as it could affect the diagnostic yield while it is a mainstay in the treatment of vasogenic edema ( Hoang-Xuan *et al*., 2015). Interestingly in our case, the initiation of high-dose dexamethasone did not affect our diagnosis. The typical diagnostic workup for CNS lymphoma consists of CSF analysis for markers such as *IL-10*, *CXCL13*, *CD19, CD20* or flow cytometry ( Baraniskin *et al*., 2011; Baraniskin & Schroers, 2014; Muñiz *et al*., 2014; Rubenstein *et al*., 2013). Due to the mass effect that is exerted by meningiomas, CSF analysis is difficult without a craniotomy as a lumbar puncture would not be recommended in such a setting. MRI is the gold standard diagnostic modality for meningiomas, however, this is complicated by the fact that CNS lymphoma can mimic any and every intracranial pathology, making it difficult to discern whether lymphoma should be considered as a possibility ( Bühring *et al*., 2001; George *et al*., 2007; Kulkarni *et al*., 2012).

The most common association of two primary intracranial tumors is that of meningioma and glioma (>40 reported cases), however given that these tumors are two of the most common primary intracranial tumors this is thought by many to be coincidental, however associations between the two pathologies have been proposed ( Ruiz *et al*., 2015; Slowik & Jellinger, 1990; Suzuki *et al*., 2010; Zhang *et al*., 2018). In a report of two patients with concurrent meningioma and high grade gliomas, Ruiz *et al.* reported a mutation in *K409Q* of the *KLF4* gene within the meningiomas ( Ruiz *et al*., 2015). Suzuki *et al.* reported an oncogenic effect due to overexpression of platelet-derived growth factor (PDGF) receptors ( Suzuki *et al*., 2010).

Simultaneous presentations tend to affects adults and have a female predominance due to the nature of meningiomas and their apparent relationship with progesterone and estrogen receptors ( Pravdenkova *et al*., 2006). Since meningiomas typically have an indolent course, this is likely why they are often found concurrently with another primary intracranial pathology. In the setting of simultaneous extra-axial and intra-axial lesions, primary CNS lymphoma must remain a consideration to ensure accurate diagnosis and treatment.

## Consent

The patient and her family gave written informed consent for presenting all pertinent clinical information in this case report.

## Data availability

All data underlying the results are available as part of the article and no additional source data are required.
