# Supplementary material for: Case Report: Concurrent primary CNS lymphoma and meningothelial meningioma - nuances of diagnosis and management
Source: F1000Res. 2019 Aug 28;8:103. Originally published 2019 Jan 25. [Version 2] doi: 10.12688/f1000research.17770.2 (PMC11809638; doi:10.12688/f1000research.17770.2)
Supplement: Peer review history

# Reviewer report: Reviewer response for version 2

**DOI:** 10.5256/f1000research.22421.r351183

**Published:** 2025-01-02

**Authors:** Mustafa Ismail

**Affiliations:** 1 University of Baghdad, Baghdad, Iraq

This case report effectively highlights the rare simultaneous presentation of meningioma and primary CNS lymphoma, but it could be significantly enhanced by addressing gaps in diagnostic, surgical, and follow-up details. A more comprehensive preoperative workup, including CSF analysis, PET-CT, and bone marrow biopsy, is essential for ruling out systemic lymphoma and ensuring accurate staging. Neurocognitive evaluation before and after treatment could provide valuable insights into patient outcomes. The surgical details should include the exact approach to the periventricular lesion and the use of neuronavigation or intraoperative monitoring, if any. Additionally, incorporating specific details about chemotherapy and radiotherapy regimens, such as doses and cycles, would provide a clearer picture of the treatment plan. Addressing these elements would strengthen the report’s educational and clinical value.

 To further enhance the discussion, the authors could compare this case’s outcome with the other nine reported cases of concurrent meningioma and CNS lymphoma, offering a broader perspective on prognosis and management. Exploring potential pathophysiological links, such as inflammatory pathways or genetic mutations, would enrich the scientific understanding of this phenomenon. Furthermore, emphasizing the critical role of timely translation services in avoiding diagnostic delays and summarizing key clinical takeaways, such as prioritizing imaging modalities and surgical approaches for co-occurring tumors, would add practical insights for clinicians.

Are enough details provided of any physical examination and diagnostic tests, treatment given and outcomes?

Yes

Is the case presented with sufficient detail to be useful for other practitioners?

Partly

Is sufficient discussion included of the importance of the findings and their relevance to future understanding of disease processes, diagnosis or treatment?

Partly

Is the background of the case’s history and progression described in sufficient detail?

Partly

Reviewer Expertise:

Neurosurgery, Endovascular Neurosurgery,

I confirm that I have read this submission and believe that I have an appropriate level of expertise to confirm that it is of an acceptable scientific standard, however I have significant reservations, as outlined above.

# Reviewer report: Reviewer response for version 2

**DOI:** 10.5256/f1000research.22421.r53077

**Published:** 2019-09-11

**Authors:** Kuntal Kanti Das

**Affiliations:** 1 Department of Neurosurgery, Sanjay Gandhi Postgraduate Institute of Medical Sciences, Lucknow, Uttar Pradesh, India

The paper is acceptable to me.

Are enough details provided of any physical examination and diagnostic tests, treatment given and outcomes?

Yes

Is the case presented with sufficient detail to be useful for other practitioners?

Yes

Is sufficient discussion included of the importance of the findings and their relevance to future understanding of disease processes, diagnosis or treatment?

Partly

Is the background of the case’s history and progression described in sufficient detail?

Partly

Reviewer Expertise:

NA

I confirm that I have read this submission and believe that I have an appropriate level of expertise to confirm that it is of an acceptable scientific standard.

# Reviewer report: Reviewer response for version 1

**DOI:** 10.5256/f1000research.19429.r51975

**Published:** 2019-08-20

**Authors:** Kuntal Kanti Das

**Affiliations:** 1 Department of Neurosurgery, Sanjay Gandhi Postgraduate Institute of Medical Sciences, Lucknow, Uttar Pradesh, India

The authors report an interesting case of co-occurrence of primary CNS lymphoma and meningioma in a 64-year-old lady and discuss the importance of preoperative suspicion in their management. The case is indeed interesting and clearly, the authors managed the case well. 

 My observations which the authors may like to clarify include:

1. Was the presence of hemiparesis not suspected when the patient presented with a fall 2 weeks earlier? Clearly, the tendency to fall on the left side must have been given due importance.
2. Details of the surgical approach are necessary. How was the periventricular lesion approached? Through a separate corticectomy or through the bed of the meningioma cavity? Was any intraoperative image guidance utilized?
3. Did you try to rule out systemic lymphoma? If so, how?
4. Apart from being a function of increased age, is there any connecting link between lymphoma and meningioma? Some more literature needs to be incorporated in this regard. 

Apart from reporting a rare association, a case like this can be more educative if newer insights can be provided as to how do they co-occur and how should they be managed. I would be happy if the authors address these minor points.

Are enough details provided of any physical examination and diagnostic tests, treatment given and outcomes?

Yes

Is the case presented with sufficient detail to be useful for other practitioners?

Yes

Is sufficient discussion included of the importance of the findings and their relevance to future understanding of disease processes, diagnosis or treatment?

Partly

Is the background of the case’s history and progression described in sufficient detail?

Partly

Reviewer Expertise:

Neurooncology

I confirm that I have read this submission and believe that I have an appropriate level of expertise to confirm that it is of an acceptable scientific standard, however I have significant reservations, as outlined above.

# Reviewer report: Reviewer response for version 1

**DOI:** 10.5256/f1000research.19429.r49515

**Published:** 2019-06-17

**Authors:** Eric Bessell

**Affiliations:** 1 Department of Clinical Oncology, Nottingham City Hospital, Nottingham, UK

The authors are correct in stating that a concurrent presentation of meningioma and primary CNS lymphoma (PCNSL) is very rare and worth publishing. The authors imply that the use of translators for the hispanic population in California is not routine. They should clarify this for an international audience. PCNSL can be rapidly progressive and urgent investigation is essential. 

 Most cases of PCNSL are diagnosed by stereotactic biopsy +/- CSF cytology and a surgical approach is uncommon. In this case the radiological appearance was of a meningioma as well as a lesion adjacent to the right postero-lateral ventricle. The frozen section of the latter lesion suggested a high-grade glioma and for this reason surgical removal of both tumours was indicated.

 No detailed information is given on the staging investigations carried out which should include CT or PET/CT scanning, CSF cytology, bone marrow aspirate and trephine, slit-lamp examination of the eye and blood tests including LDH. Also baseline neuro-cognitive tests are indicated at the age of 64 years.

 No detailed information is given on the chemotherapy given, the regimen with methotrexate, cytarabine and rituximab is not referenced and no doses are given and the number of cycles not stated. In addition the radiotherapy dose is in Gy with the number of fractions stated not in cycles. This information is essential in a patient of this age.

 The authors state that at 2 years there is no recurrence but again give no indication of whether there has been any cognitive deterioration.

 In the discussion the authors discuss the other brain tumours that occur concurrently with meningioma but don't discuss the outcome achieved in the other 9 cases reported and how they compare with the case they are reporting.

 In summary this is an interesting case report which would be greatly enhanced if the additional information I have alluded to was included.

Are enough details provided of any physical examination and diagnostic tests, treatment given and outcomes?

Partly

Is the case presented with sufficient detail to be useful for other practitioners?

Partly

Is sufficient discussion included of the importance of the findings and their relevance to future understanding of disease processes, diagnosis or treatment?

Yes

Is the background of the case’s history and progression described in sufficient detail?

Yes

Reviewer Expertise:

International expert in primary CNS lymphoma and co-author of European Guidelines in 2015.

I confirm that I have read this submission and believe that I have an appropriate level of expertise to confirm that it is of an acceptable scientific standard, however I have significant reservations, as outlined above.
